# Bilateral ulna hemimelia with humeroradial synostosis and oligodactyly: A case report

**DOI:** 10.1016/j.radcr.2024.02.056

**Published:** 2024-03-15

**Authors:** Yaa Achiaa Afreh, Kwasi Adjepong Twum, Adu Tutu Amankwa, Kwasi Ankomah, Obed Kojo Otoo, Caroline Oku

**Affiliations:** a Radiology Directorate, Komfo Anokye Teaching Hospital (KATH). P.O. Box 1934, Kumasi, Ghana; b Department of Radiology, School of Medicine and Dentistry, Kwame Nkrumah University of Science and Technology (KNUST), Private Mail Bag, University Post Office, Kumasi, Ghana

**Keywords:** Ulna, Hemimelia, Micromelia, Humeroradial synostosis, Oligodactyly

## Abstract

Hemimelia denotes the partial or complete absence of the distal half of a limb. Ulna hemimelia, a rare congenital anomaly, involves the complete or partial absence of the ulna in the upper limb, with an incidence of 1 in 150,000. This condition has been classified into 4 types, with the rare Type 4 variant involving humeroradial synostosis. We present a unique case of bilateral complete ulna hemimelia, humeroradial synostosis, and oligodactyly, in an 11-month-old female with bilateral upper limb shortening and restricted elbow movement since birth. Clinical examination revealed bilateral upper limb shortening, medial deviation of both wrist joints, fixed extension of both elbow joints, and bilateral absence of the cubital fossa. Radiographs confirmed bilateral micromelia, absence of ulna, humeroradial synostosis, and oligodactyly. This case, exhibiting bilateral Type 4 ulna hemimelia with Class 1 humeroradial synostosis, is a complex variant, rarely reported, and the first documented in Ghana. It also highlights the importance of radiological assessment in ensuring accurate diagnosis. Long-term follow-up and potential surgical interventions are crucial for optimizing upper limb function in such cases.

## Introduction

The term “hemimelia” was coined by Isidore Geoffroy Saint-Hilaire in 1836 and refers to the partial or complete absence of the distal half of a limb [1]. Ulna hemimelia is characterized by a postaxial longitudinal deficiency in the upper limb, where the ulna is either completely or partially absent [1].

Initially documented in 1683, this congenital anomaly is rare, with an incidence of 1 in 150,000 [1]. Males are more commonly affected, exhibiting a male-to-female ratio of 3:2 [2]. Approximately 70% of cases are unilateral, often on the right side, and tend to be incomplete [2,3]. While some individuals may remain asymptomatic with mild isolated ulna deficiency, complex ulna deficiencies can lead to various upper limb abnormalities, such as humeroradial synostosis, radial head dislocation, metacarpal and carpal coalition, and digital abnormalities [1]. Given the diverse nature of forearm and hand deformities, radiological investigations are crucial for accurately identifying the specific type of deficiency [1].

Although a limited number of cases have been documented in the literature [4], [5], [6], [7], [8], we present a unique instance of bilateral complete ulna hemimelia accompanied by humeroradial synostosis and oligodactyly.

## Case presentation

An 11-month-old female child presented with bilateral upper limb shortening and limited elbow movement from birth. Mother was not a regular antenatal clinic attendant and pregnancy was poorly monitored. Nevertheless, she delivered in a health facility. There was no history of drug or alcohol abuse during pregnancy.

**Fig. 1:**
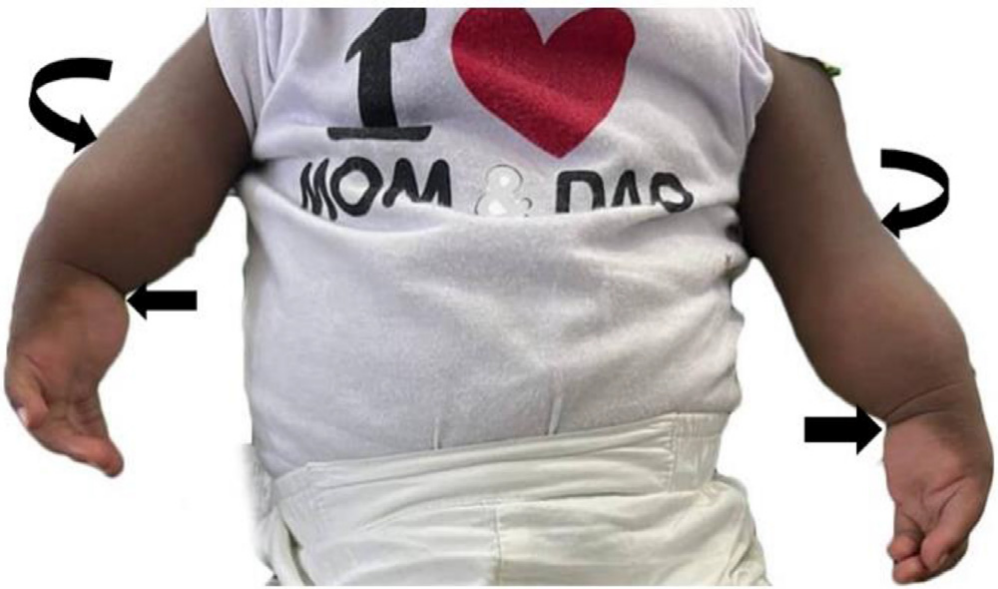
Clinical photograph of patient showing bilateral upper limb shortening and extension, absence of cubital fossae (*curved arrows*) and medial deviation of the wrist joints (*block arrows*).

**Fig. 2:**
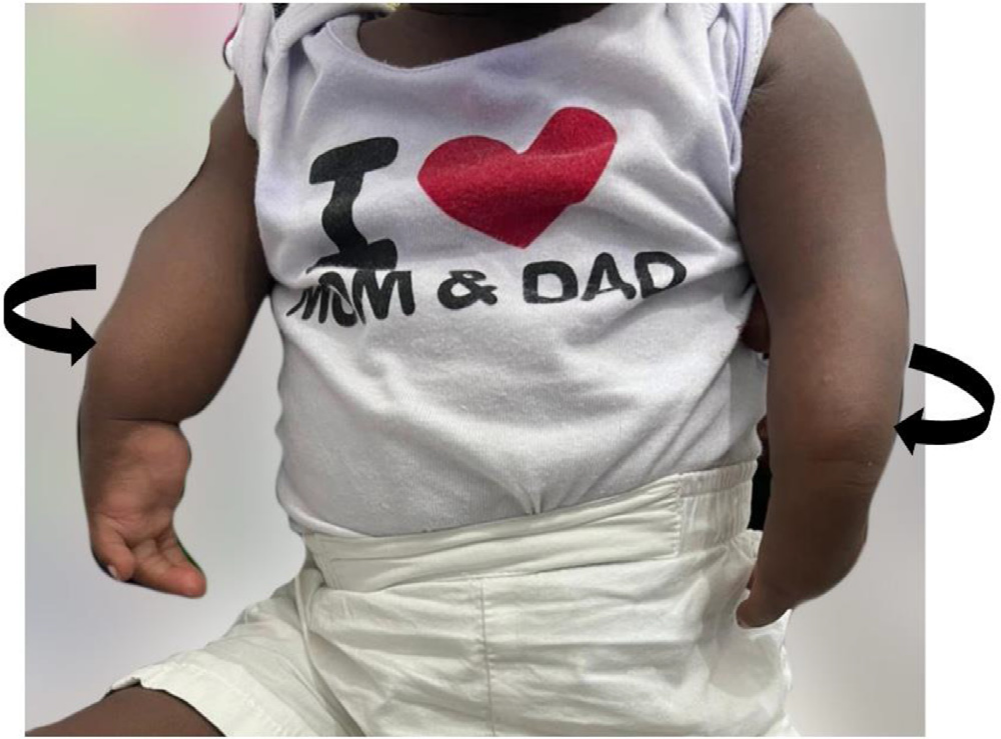
Clinical photograph showing internal rotation of both upper limbs (*curved arrows*).

Upon examination, bilateral upper limb shortening with medial deviation of both wrist joints was observed (Fig. 1). While shoulder and wrist movements were within normal limits, there was a fixed extension of the elbow joints bilaterally, and no elbow joint movement was noted. Both upper limbs were internally rotated, and the cubital fossa was absent bilaterally (Figs. 1 and 2). No other major anomalies were evident. Radiographs of the vertebral column revealed no abnormalities, and both echocardiography and renal ultrasound results were within normal limits.

**Fig. 3:**
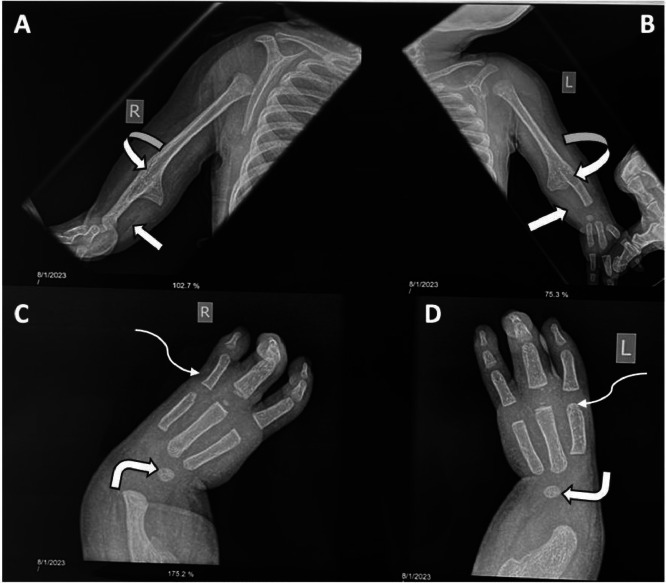
(A-D): Antero-posterior radiographs of both upper limbs (A and B) showing absence of the ulna bilaterally (block arrows) with elbow fixed in extension and bilateral humeroradial bony fusion (curved arrows). Anteroposterior radiographs of both wrists (C and D) show ulnar deviation with the presence of one carpal bone (bent arrows) and three digits (tridactyly) (squiggle arrows).

Anteroposterior radiographs of both upper limbs indicated bilateral micromelia, with the absence of the ulna bilaterally and shortening of the radii. The upper limbs were fixed in an extended position, and bilateral humeroradial synostoses were also present (Fig. 3 A and B). Additionally, anteroposterior radiographs of both wrist joints and hands revealed ulna deviation of the wrist joints bilaterally, with only one carpal bone visible per wrist. Furthermore, both hands displayed 3 digits (Fig. 3 C and D).

## Discussion

Ulna hemimelia is a rare congenital upper limb deficiency resulting from the incomplete formation of part or the entire ulna. The rarity of this deformity is attributed to the early development of the ulna during the embryonic period (24-36 days), corresponding to a period of high fetal mortality [9]. While often nonsyndromic, ulna hemimelia may be associated with conditions such as Poland syndrome, Goltz-Gorlin syndrome, Cornelia de Lange syndrome, or femur fibula ulna syndrome [10].

Bayne et al. [11] classified ulna hemimelia into 4 types; Type 1 refers to hypoplasia of the ulna with both the proximal and distal epiphysis being present, Type 2 partial aplasia of the ulna, Type 3 is complete absence of the ulna that is accompanied by severe carpal and digital abnormalities, and Type 4 including humeroradial synostosis.

Humeroradial synostosis, a rare condition [6], can be further classified into Class 1 (sporadic, fixed in extension with ulna ray hypoplasia) and Class 2 (familial, fixed in flexion without hypoplasia) [12]. Pffeifer and Braun Quentin also studied the genetic basis of humeroradial synostosis and categorized it into three entities, autosomal dominant ankylosis of the elbow with multiple joint synostosis, autosomal recessive humeroradial synostosis with ulna dysgenesis and possibly the femur and fibula but without digital anomalies and nongerminal humeroradial synostosis as part of ulna longitudinal dysplasia with digital anomalies [13].

Bilateral ulna hemimelia and bilateral humeroradial synostosis are uncommon, with previous studies reporting bilateral ulna hemimelia in 35.3% [4] and bilateral humeroradial synostosis in 21% [8] of cases. Our case of bilateral Type 4 ulna hemimelia with Class 1 bilateral humeroradial synostosis is a rare and complex variant, potentially the first reported in Ghana. Notably, our female patient contradicts the usual male prevalence [2], similar to what Aggarwal et al. [6] have documented, and she falls into the Pfeiffer and Braun Quentin Type 3 category, the most common in a study by El-Hassan et al, accounting for 92.8% of cases [8].

Ulna hemimelia may also be associated with complex carpal, metacarpal, and digital abnormalities. Aplasia, hypoplasia and fusion of the carpal or metacarpal bones may be present with the triquetrum and capitate being frequently absent in these patients [1]. Our case exhibited one carpal bone in the wrists bilaterally. Accounting for the age (the capitate and hamate ossification centers are expected to be present), this suggests absence of the capitate. Furthermore, our patient exhibited tridactyly (a three-fingered hand), the predominant hand anomaly linked to ulna hemimelia, with monodigital hand being the subsequent occurrence [1,5].

Although humeroradial synostosis can be associated with Antley-Bixter syndrome, a rare disorder characterized by various manifestations including humeroradial synostosis, camptodactyly, arachnodactyly, joint contractures, craniosynostosis, brachycephaly, dysplastic ears and midface hypoplasia, our patient did not exhibit other associated features. Though our patient had bilateral humeroradial synostosis we did not suspect Antley-Bixter syndrome since the other manifestations were absent.

Management of these conditions is challenging due to the complexity of abnormalities, requiring tailored approaches. Treatment typically involves passive physiotherapy to prevent disuse atrophy. Unilateral ulna hemimelia often does not require surgical intervention [5] as most patients are able to perform activities of daily living without significant restrictions [8], while bilateral cases may necessitate procedures such as soft tissue releases, humeral derotational osteotomies, or elbow disarticulation with prosthetic placement [5]. In our case, continuous passive physiotherapy was initiated, and long-term follow-up is planned to assess the need for surgical intervention and monitor overall progress.

## Patient consent

A written informed consent relating to use of patient's medical history and radiological images was obtained after careful explanation to the patient's guardian that their anonymized images and clinical history will be used for publication in a scientific journal.
